# The place of learning in a universal health coverage health policy process: the case of the RAMED policy in Morocco

**DOI:** 10.1186/s12961-019-0421-6

**Published:** 2019-02-21

**Authors:** E. Akhnif, J. Macq, Bruno Meessen

**Affiliations:** 1 School of Public Health, Rue Lamfadel Cherkaoui, Madinat Al Irfane, BP-6329 Rabat, Morocco; 2 IRSS - Clos Chapelle-aux-champs 30 bte B1.30.13 à 1200 Woluwe-Saint-Lambert, Université Catholique de Louvain (UCL) Ottignies-Louvain-la-Neuve, Brussels, Belgium; 3 Institute of Tropical Medicine, Antwerp, Belgium; 4 Community of Practice ‘Performance-Based Financing’, Antwerp, Belgium

**Keywords:** Universal health coverage, health system, learning organisation, health financing

## Abstract

**Background:**

To progress towards universal health coverage (UHC), each country will have to develop its systemic learning capacity. This study aims at documenting how, across time, learning can feed into a UHC policy process, and how the latter can itself strengthen (or not) the learning capacity of the health system. It specifically focuses on the development of a major health financing policy aligned with the UHC goal in Morocco, the RAMED, a health financing scheme covering hospital costs for the poorest segment of the population.

**Methods:**

We conducted a retrospective analysis of the RAMED policy for the period between 1997 and 2018, along with a case study design. For the data collection and analysis, we developed a framework combining Garvin’s learning organisation framework and the heuristic health policy analysis framework. We gathered data from key informants and document reviews.

**Results:**

The study confirmed the importance of learning during the different stages of the RAMED policy process. There is evidence of a leadership encouraging learning, the introduction and adoption of knowledge management processes, and the start of a transformation of the administrative culture. Yet, our study also showed some major shortcomings, especially the lack of structure of the learning, and insufficient effort to systemise and sustain a transformation of practices within the health administration. Our study also confirms that the learning changes in nature across the different stages of the policy process.

**Conclusion:**

The policy decisions and the implementation strategy create a learning dynamic, though not structured in all cases. Despite the positive interaction between learning and the RAMED policy, the opportunity to push forward a more structural transformation towards a learning system has not been fully seized. Hierarchical logics still largely prevail in the Moroccan health administration. The impact of future health policies for both the target beneficiaries and the health system will be bigger if their design integrates purposeful and structured actions in favour of organisational learning. This recommendation probably applies beyond Morocco.

**Electronic supplementary material:**

The online version of this article (10.1186/s12961-019-0421-6) contains supplementary material, which is available to authorized users.

## Key messages

- Country systemic learning capacities will be key to progress towards Universal Health Coverage.
- Learning capacities enhance policy development, but can also benefit from the latter.
- Learning should receive more attention in health policy analyses.

## Background

Universal health coverage (UHC) is a topic of concern at the global level. It is defined as the capacity to provide all people with access to health services of sufficient quality, while also ensuring that the use of these services does not expose the user to financial hardship [1]. In many low- and middle-income countries (LMICs), progress towards UHC will require strengthening of the health system [2, 3] and the introduction and rollout of medical coverage schemes for under-covered groups such as very poor households or people working in the informal sector [4].

A growing number of scholars and actors are studying the path to UHC [5]. A recent study looked at 24 countries and highlighted the existence of diversity in paths in terms of strategic choices that led to different results [6]. In fact, for any country, the road to UHC is strongly linked to the complex process by which policy decisions take place. Thus, the transferability of experiences from one country to another is deemed to be somewhat limited [5, 6], wherein each country will have to find its own way to reach UHC. This statement suggests a key recommendation to countries, namely that to progress towards UHC, each country must develop its capacity to learn from its own experience [7]. However, so far, how to develop systemic learning capacities has been given little importance by the health sector actors, especially in LMICs [8].

For this research programme at the crossroads of UHC, policy and learning processes, the research community does not start from scratch. For instance, the heuristic stage framework [9] pays particular attention to the stage of monitoring and evaluation, and acknowledges the feedback loop between the policy and the emerging learning. There is also a huge literature on the uptake of evidence to inform UHC policies [10, 11], together with a growing recognition that learning should not stop at research findings [12]. More recently, scholars have started to look at health systems as complex systems not complying with deterministic causal models [13, 14]. A recommendation emerging from this recognition is that investing in learning capacities is key [15]. It has also been recommended to adopt new ways of thinking to close the knowledge-action gap; innovation in health systems should constitute learning opportunities [16].

The importance of looking at learning processes in health financing development in a comprehensive way is also attracting more attention. Some health policy analysts have tried to look at how knowledge contributes to shaping and affecting health financing policies [17–19]. For instance, Ir et al. [20] used a knowledge translation framework to analyse the development of health equity funds in Cambodia. Their study shows how lessons from the pilot experience helped feed into national policy. More recently, Meessen et al. [21] put knowledge as one of the key dimensions where progress should take place during the scale-up process of a health financing strategy, with the recognition that this progression is itself multidimensional (e.g. from hypothesis to evidence, from theory to practice, from a few persons to many) and benefits from purposeful processes (e.g. experimentation via pilot projects). However, this does not say much on how to structure learning capacities within the health system, and the administration in particular.

It is only very recently that health system researchers have realised that they should tap the important body of knowledge developed on organisational learning in business studies [7]. Organisational learning has been described in several ways. It is said to be the cumulative product of learning in small groups or teams [9]. It determines the capacity for organisations to learn from experience and to exploit the knowledge of others to contribute to organisational intelligence [22]. Organisational learning is also defined as the collective learning triggered in an organisation by creating a capacity to impact its performance [23, 24]. Sharing knowledge that remains in the organisation, regardless of changes in healthcare teams or members, contributes to organisational effectiveness and efficiency [25]. A learning organisation can then be defined as an organisation where conditions for organisational learning are in place [26].

This study aims at documenting how, across time, learning can feed into a UHC policy process, and how the latter can itself strengthen (or not) the learning capacity of the health system. Despite weaknesses in terms of learning at the level of the Moroccan health system [27], the RAMED (*Régime d’Assistance Médicale*), a health coverage scheme for the poor, provides an interesting case – it is indeed considered one of the structuring policies that contributed to health system development and allowed Morocco to make significant progress towards UHC (9 million were covered by this scheme in 2015) [28]. Furthermore, the fact that several LMICs (e.g. Thailand, Cambodia and Mexico) made important progress towards UHC by the rollout of a scheme targeting the (important) poor segment of their population and that a country like India is about to take the same road at a massive scale [29] indicates that the RAMED policy is of interest beyond the strict case of Morocco.

The objective of this research is to focus on the nature of an assumed bi-directional relationship between policy development and organisational learning. The two research questions focus on assessing how pre-existing (organisational) learning contributed to the development of the RAMED policy and how the RAMED policy contributed to strengthening the attributes of organisational learning within the Ministry of Health of Morocco.

### The financing of the Moroccan health system and RAMED

According to the latest Moroccan national health accounts, the total health expenditure in 2013 reached approximately 52 billion dirhams ($US 6 billion at the 2013 exchange rate), amounting to nearly $US 188 per capita. This total health expenditure represents 5.9% of the GDP. The sources of financing of the health system are tax revenue (24.4%), households (50.7%), health insurance (22.4%), employers (1.2%), and international cooperation and others (1.3%). The scale of the solidarity is thus still limited, with the most significant part of health financing being out-of-pocket.

The main objective of the RAMED scheme is to provide financial protection for the poor and the near poor in their use of public hospitals. For enrolment, an identification system based on ‘means scores’ was developed. The RAMED is mainly subsidised by the government through resources allocated to the Ministry of Health (MoH). The RAMED started with a pilot experiment in 2008 and was generalised in 2012. Today, it covers 28% of the population.

## Methods

We used a case study as the design for this research. We conducted a retrospective analysis of the RAMED policy for the 1997–2018 period using a specific framework and collecting data from key informants and document reviews.

### Conceptual framework

**Fig. 1:**
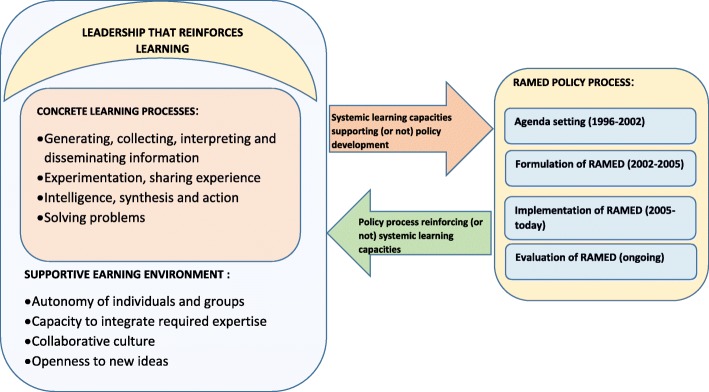
Our conceptual framework

For our analysis focused on learning, we have opted for an adapted version of the framework developed by Garvin et al. [30, 31], which is one of the most commonly used in the health system literature [8]. We have used and appreciated its power in a previous cross-country empirical study [7]. Our adapted version of the framework is organised around Garvin’s three main blocks, namely (1) leadership reinforcing learning (encourages the use of knowledge from learning and practice); (2) an environment supportive of learning (space for new ideas to emerge, to be tested, analysed and discussed collectively before adoption and scale-up); and (3) practical processes for learning (sustained and systematic mechanisms of the production of learning and knowledge) (Fig. 1).

We have combined this framework with the so-called ‘stage heuristic framework’ [9, 32], which structures a health policy into four main sequential stages, namely agenda-setting, policy formulation, policy implementation, and policy evaluation [33]. This sequential approach to the policy process helps with both the description and the analysis of what happened.

Our conceptual framework therefore emphasises the interrelation between the organisational dynamic and policy development. However, our choice of focusing on the learning has an opportunity cost – in our analysis, we had less space to discuss the other dimensions of the policy process such as the context and the role of actors [34].

#### Source and methods for data collection

For the data collection, we designed a grid that takes into account the elements of learning as explained in our framework and allows analysis of each of the stages of the RAMED policy. This led to a semi-structured questionnaire used for the interviews and a tool for document data extraction. The questions in our questionnaire were inspired by a survey tool developed for our previous cross-country analysis [7].

To answer our two main objectives, we organised our documentation of the RAMED policy process into four sub-research questions, as follows:

1. What are the factual elements that demonstrate effective learning for a better RAMED policy? What are their characteristics? At what level of the health system did these different learnings take place?
2. How did learning occur during the major phases of policy development? Did learning take different forms for the different heuristic stages of the policy? What mechanisms and routines have been implemented to ensure learning?
3. What were the barriers or facilitating factors of learning for the RAMED policy, at the level of the actor (including leadership), context (including organisational culture), process and content?
4. Is it possible to identify mechanisms or processes by which the development of the RAMED policy itself would have led to a strengthening of the health system capacity to learn? (progress towards the learning organisation).

#### Document review

In order to understand the RAMED policy elements we conducted a documentation review [35–42]. To identify the more important documents, we set the selection criteria of including the documents most used by decision-makers and those most recommended by actors involved in the policy implementation; we also used our knowledge of the policy to choose the most relevant documents. We searched these documents through the websites of the National Agency for Health Insurance, the National Observatory of Human Development and the reports available in the archives of the MoH and of financial and technical partners (WHO, European Union, World Bank, etc). We also contacted resource personnel at the MoH. The document review contributed to shaping the timeline of the policy (Additional file 1) and helped to give a documented introduction to each phase of the policy.

#### Key informant interviews

For our case study, data collection was also based on semi-structured interviews. The key informants were selected among people who had participated in at least one phase of the RAMED policy and had played an important role in this process. For the agenda-setting and policy formulation, people at the central level were interviewed; for the implementation of RAMED, in addition to people at the central level, there were also people from the regional and local levels of the MoH. Given the cross-sectoral nature of the policy, a few people outside the MoH were also interviewed. The sample was determined according to the importance of each phase (number of years and complexity of the phase); we started with a number of interviewees and continued recruiting until saturation was achieved for the phase.

The interviews were conducted by the first author. All our interviewees agreed to conduct the interviews in French, agreed to be recorded and gave verbal consent. The duration of each interview varied from 1 to 1.5 h.

Table 1 describes the sample of the study according to the profile of the interviewees and the stage of the policy in which they were involved. For the policy formulation, we interviewed three people from the MoH who were involved in the inter-ministerial committee; we noticed from the second interview that they shared very similar answers as they had worked in the same group. The policy implementation took place from 2008 to 2018 and still continues; therefore, we devoted a great number of interviews to this phase to capture all aspects and achieve saturation.

**Table 1:** Description of participants’ profiles (*n* = 18)

Policy stages	Senior officials, high level decision- makers	Heads of department or divisions involved in RAMED policy (including one from the Ministry of Finance)	Head of services	Regional director	Hospital director	Technical and financial partners	National observatories’ researchers	Senior advisers on UHC	Senior officials of national health insurance bodies
Agenda-setting and policy formulation	1	2							
Policy implementation	1	3	2	1	2			1	1
Policy evaluation						2	2		

#### Data analysis

After conducting and recording all interviews, we transcribed them into Word documents. We then coded the transcripts based on the elements of our framework using NVivo to organise our codes and an Excel sheet to structure our summary of the results.

The document review provided an introduction documenting each phase in the result section. It also allowed us to finalise the timeline (Additional file 1).

#### Ethical considerations

The study protocol was approved by the ethical committee of the Institute of Tropical Medicine of Antwerp in Belgium (decision number 1212/18). Prior to each interview, the first author presented the objectives of the study and obtained verbal approval and consent to record the interview.

## Results

We have structured the presentation of our results by stages of the policy (agenda-setting and policy formulation, implementation and evaluation). Agenda-setting was merged with policy formulation because the focus of this article is mainly on learning within the MoH; the agenda-setting was actually very political, with a limited role for the MoH. For each stage of the policy, we present the results according to the three blocks of the Garvin framework. There are, of course, relationships between the three blocks, so some phenomena are reported in more than one block for different purposes.

### The agenda-setting and policy formulation phase (1996–2002)

The history of basic medical coverage in Morocco dates back to the 1990s (see timeline in Additional file 1). Prior to that, the poor had access to health services through so-called ‘indigence certificates’. The first draft on compulsory health insurance was formulated between 1990 and 1992, and was followed by an announcement of the principles of the basic medical coverage in a royal speech in 1993. In 1995, the government proposed the law of compulsory health insurance (*Assurance Maladie Obligatoire*; AMO) for formal employees in the private and public sectors. However, this law was not approved by parliament (at that time, the opposition was dominated by the socialist party) because it did not foresee coverage for the poor. Between 1996 and 1997, an inter-ministerial commission was created to co-produce a draft of the basic medical coverage law in Morocco. The political transition of this period (1998) [43] contributed to prioritising RAMED.

### How the policy process contributed to strengthening the learning elements

#### Leadership that reinforced learning

The first stage of the policy was characterised by direct involvement of the Prime Minister in strategic discussions on technical proposals of the policy. This high-level involvement in the policy played a role in stimulating a leadership dynamic in the ministries involved in the inter-ministerial committee and, more importantly, contributed to reducing divergences between ministries. The need to have creative ideas pushed policy-makers to encourage learning within their teams. In this sense, the leadership of the MoH was strengthened by its involvement in this committee and contributed to value learning for RAMED. The ministers and senior officials involved in the policy formulation encouraged their teams to work hard, gather knowledge and search for the best ideas. For example, teams involved in RAMED were sent to visit other countries like France, Belgium, Tunisia, Algeria and Latin American countries to learn from their experiences to make proposals for the case of Morocco.

“*The Prime Minister… introduced a learning dynamic on RAMED and he himself raised relevant questions during the discussions … in 1998, the Prime Minister himself chaired the RAMED meetings and decisions were taken immediately during these meetings on the types of study to be initiated*” (Informant 3).

“*High-level meetings were organised to discuss the options, including political leaders: there was a high political commitment*” (Informant 4).

#### An environment supportive of learning

The dynamic that was created within the inter-ministerial committee contributed to the emergence of an environment supportive of learning. The policy formulation process, although imposed by the rules of the administration and hierarchy, had some positive results in creating a collaborative dynamic. As it was a new policy, it gave importance to the use of knowledge and factual data. Individuals from each department had some autonomy in proposing new ideas, although not all were accepted. The inter-ministerial committee integrated expertise from the country, but also involved experts from other countries in consultancies. Additionally, members of the committee were obliged to work in teams and groups to ensure a ‘complementarity’ in the reflection. A culture of structured and documented meetings was adopted, which was also a way of formalising the commitment of each department to the policy. The study visits that were organised contributed to creating an openness to new ideas from other contexts. The dynamic also created informal relations between members of the committee that went beyond the hierarchy which enhanced the learning.

“*RAMED has contributed to better individual and team-level development, but lacked work at the institutional level*” (Informant 2).

For the technical work that was performed within the government departments (inter-ministerial group), the effect of hierarchy played a positive role in reducing resistance from some departments who opposed some technical choices, especially the Ministry of Finance.

“*More and more, RAMED has contributed to the development of a collaborative culture between actors and departments involved in the policy*” (Informant 1).

#### Concrete processes for learning

The policy decisions at a high level in the organisation of the formulation process created and improved some practical processes of learning, for example, internalising the environment experience through study tours in different countries; this enhanced the knowledge of individuals involved in the policy. The practice of structured meetings and sharing the minutes was also a practical process for learning that characterised this phase (generating knowledge). Universities were also involved in this dynamic through research projects concerning specific issues related to the policy (collecting, interpreting and disseminating information). In this period, resources were available for departments to develop processes for learning. Learning from experience in the field and experimentation was also developed through the testing of ideas in the field prior to presenting them in high-level meetings and taking decisions (experimentation). For example, before formulating the eligibility criteria, a series of tests were carried out by members of the committee in the field.

“*We had scholarships to develop research related to the policy and even had the opportunity to publish articles*” (Informant 1).

#### How the learning dynamic contributed to developing the policy

The learning dynamic created by the policy formulation process had as an objective the development of a draft of the law submitted for government and parliament approval. The learning initiated by members of the committee strengthened the capacity of policy-makers regarding the content of UHC. This helped to defend the draft of the law through the complex government and parliament approval process. As stated by one member of the committee:

“*In the beginning, during the technical meetings of the committee when I talked about adverse selection, people didn’t know what I was saying, but now there are many people who know a lot about the policy*” (Informant 1).

Many interviewees acknowledged that the learning dynamic through teamwork and the collaborative culture improved the quality of the technical dimension of the draft. For example, thanks to the learning and group discussions, members of the committee agreed that, in order to increase access, there should be no user fees for RAMED beneficiaries. In this example, we see clearly that learning from each other (teamwork) and the exchange of technical arguments can lead to positive decisions.

“*The discussions between the members of the committee led to ideas for the benefit of the beneficiaries of the RAMED. For example, in the beginning, some members wanted to establish a user fee for the beneficiaries, but the consensus was to remove it to improve access for the poor*” (Informant 2).

### The implementation phase of RAMED (2008–today)

After the adoption of Law 65–00 [44] in 2002, with its two components AMO and RAMED, all political attention focused on the AMO scheme, which had much stronger political backing (unions and formal sector employees). The implementation of the RAMED component was delayed until 2008, the decision to start with a pilot experiment was taken, and which eventually took a little longer than planned (almost four years, 2008–2011) [35–37]. From 2008, the government developed the regulations of the RAMED [45], which continued to evolve until 2015. In February 2011, the Arab Spring [46] struck Morocco, with the ‘February 20’ social movement [47]. The King reacted swiftly with a constitutional reform in 2011 that granted more power to the government and declared the right to access to health services for all the population. In 2012, the King decided to generalise the RAMED. The generalisation came at a time when the first evaluations of the pilot experiment had taken place in 2010 [35]. In 2013, a new inter-ministerial committee was set up for the follow-up of the scheme’s generalisation. In 2016, the inter-ministerial committee produced an interdepartmental action plan for medical coverage including the RAMED scheme. In 2017, the same committee examined the decision to create an independent body for management of the RAMED resources and regulations.

This stage of RAMED was considered by our interviewees as the phase where the learning was the most important and concerned a large number of people, unlike the formulation phase, which was restricted to a small group.

#### How the policy (implementation) contributed to strengthening the learning elements

##### Leadership that reinforces learning for policy implementation

The implementation phase was characterised by the development of leaders at different levels of the organisations involved in the RAMED policy. After the social movements of the Arab Spring, problems with access to health services were identified by the authorities as one of the triggers for social protests in Morocco. RAMED was therefore considered an issue of security and political stability across the government apparatus. This importance, and the high expectations of the population, forced leaders to be more sensitive in examining options and strategies before implementing them and so helped trigger a learning dynamic to solve problems. Indeed, as mentioned by a regional director, because of the role given to the Ministry of Interior, local governors played the role of coordination and promotion of the use of knowledge and learning within the rules of the administration. The successive Ministers of Health who managed the implementation phase also played a leadership role and promoted learning at different levels of the Ministry. Additionally, the pilot experiment was an opportunity for emerging local health leadership at the operational level such as regional directors, provincial medical officers and hospital directors who adopted and encouraged the learning dynamic.

“*The Minister of Health has formed a group of directors to reflect on basic medical coverage with working sessions even in his home*” (Informant 5).

Leadership that is supportive of learning was also encouraged and inspired by the attitude of leaders at the central level, who encouraged regional leaders to be sensitive to the use of learning. In their own words:

“*We were lucky to have a secretary general who supported us and helped us in the learning on the RAMED*” (Informant 5).

“*The governor of the Ministry of Interior involved me in many meetings which were sources of learning*” (Informant 6).

Leadership that reinforced learning was illustrated by the large number of meetings and the commission of many studies, along with the dynamic that was created at the operational level to promote learning. Thus, with the implementation of the policy, health system managers started to adopt a participative approach to making decisions.

##### Supportive environment for learning in the policy implementation phase

The interviewed actors acknowledged the importance of RAMED as an issue of national debate which generated an environment conducive to learning. Firstly, managers at different levels had some freedom and autonomy to propose ideas and strategies in meetings at the central level, even if not all ideas were taken into account. Additionally, a dynamic of exchange was created between the central and regional levels (openness to new ideas from the field). During the implementation, several consultancies involving national and international expertise were launched to develop organisational tools and procedures. This environment was illustrated by dynamic meetings and seminars, with shared reports, in which a multisectorial collaboration culture was developed. The creation of many committees, some of them strategic, contributed to sharing knowledge and the institutionalisation of teamwork. In this dynamic, managers were encouraged to innovate in relation to the strategies and solutions to problems encountered.

“*A member of my team from my department went home during a weekend to work on the eligibility criteria to develop a computer application. No one asked him to do this. It is an example of innovation*” (Informant 8).

“*The culture of sharing was there at the central level. We were always involved in bodies and committees. At the level of the region, I created evaluation committees. We collect information and create a group dynamic, and we take decisions with an improvement plan*” (Informant 5).

At the regional level, other regions created unofficial networks to learn from the experience of the pilot region through visits or phone calls to ask about how they dealt with some implementation problems (sharing experiences). Further, a dynamic of solving problems at the regional level was created with the participation of the civil society (collectively solving problems). The organisation of an international symposium to share the experience of RAMED and learn from other countries’ experiences confirmed the environment for exchange and learning [48].

##### Concrete processes for learning in the implementation phase of the policy

Experimentation was conducted through the testing of tools and organisational mechanisms in the field before the formulation of laws or strategies. The eligibility criteria for the pathway of care for RAMED beneficiaries were tested during the pilot experiment and evaluated and updated. The sharing of experiences between levels, regions or hospitals was developed, but was not systematic or integrated within health organisations’ procedures. Systematic meetings at different levels and sharing their minutes were other practical processes that contributed to developing learning for the RAMED policy.

“*We received calls from our colleagues in other hospitals to learn how to deal with problematic situations, but they were limited to friendly relationships, not initiated by the central level for all hospitals to learn from the pilot experience*” (Informant 12).

The development of cooperation projects with technical assistance (with the European Union, World Bank, WHO and African Development Bank) was also considered as a process of learning that exposed managers to other experts’ opinions and evaluations (generating, collecting, interpreting and disseminating information). The RAMED also favoured exchanges between managers of regions and hospitals, but this was not systematic and depended on the manager’s profile and motivation. The training on a large scale that accompanied the policy was considered a practical process. Additionally, the institutionalisation of an annual report about the achievement of RAMED through the creation of an observatory at the level of the hospital directorate contributed to initiating the intelligence, synthesis and action functions.

The annual national meeting to share the progress of the policy was also a practical process, because it allowed confrontation of different opinions, information, reports and critiques, and was described by implementation actors as very interesting and a rich source of learning [48]. These meetings led to the adjustment of some strategies and the correction even of regulations through amendments. For example, meetings about the RAMED eligibility criteria led to changing them based on the evaluation of the pilot experiment.

“*Individual level learning exists and it is daily. Collective sharing remains dependent on the dynamics of the local team. The major problem is the systematisation of learning*” (Informant 15).

#### How learning elements contributed to developing the policy

The learning dynamic that was created by the RAMED policy built the capacity of leadership at the regional level to make suggestions regarding the implementation of the policy. Indeed, before the policy implementation, the process of making decisions was vertical and not based on participation; this only started to change with the RAMED. The most important aspects were related to the creation of a collective problem-solving dynamic, as stated by one informant at the regional level:

“*The culture of sharing was there at the central level. We were always involved in bodies and committees. At the regional level, I created evaluation committees. We collect information and we create a group dynamic, and we take decisions with an improvement plan*” (Informant 6).

The learning environment that was created through the policy enhanced the implementation by creating a climate for developing technical guidelines and documented organisational procedures (many guidelines were edited), especially during the pilot experiment. Further, the learning dynamic within the inter-ministerial committee led to taking some strategic decisions, like creating an independent fund for RAMED resources to ensure the separation of the financing function from the service delivery function in 2017.

“*Several debates on the RAMED between all the departments on governance, the sustainability of the scheme ... etc. – the exchanges were quite positive, otherwise we would not have made the decision to go to an independent management body of RAMED*” (Informant 5).

The learning from the experimentation process, including the discussion dynamic that was created, contributed to reviewing many organisational aspects. The evidence produced urged the government to allocate resources for the infrastructure of hospitals for 5 years, although this was not enough. On the other side, the new inter-ministerial committee was created in 2013 after the lessons learnt from the first years of RAMED implementation to improve governance through an interdepartmental learning dynamic. This new dynamic led to an action plan in 2017 for the generalisation and improvement of medical coverage, including RAMED.

“*RAMED has contributed to developing a culture of producing leaflets and policy briefs, the synthesis of which has been shared through forums and meetings organised at central level*” (Informant 17).

### Organisational learning within the evaluation phase (2011–today)

Our presentation of the result does not follow the same structure as the former sections, as it was not directly led by the MoH as an activity of its organisations.

Since 2011, different evaluations have been conducted to generate lessons for the generalisation and implementation of RAMED. The first evaluation was conducted on the pilot experiment in 2010, which summarised the main achievements and pitfalls of this phase [35]. In 2013, 1 year after the scale-up, another evaluation was conducted with the support of the European Union [38]. Finally, in 2017, another evaluation, which concerned the whole policy of RAMED, was performed by the National Observatory of Human Development [39]. Additionally, a few other evaluations were carried out by other groups [49, 50].

The contribution of the evaluation phase consisted in stimulating communication between the departments involved in RAMED, and showed that the RAMED leadership was aware of the importance of learning for the policy. Further, in the evaluations, innovation was encouraged to recommend solutions for each of the problems with RAMED. To illustrate the success of RAMED, the evaluation actors mentioned that the experience of RAMED has enriched other social policies (debate on the creation of a national social registration system).

The negative aspects that remain unsolved are mainly human resources and financing of the health system. Although almost all actors agreed that the financing of RAMED has to increase, the Ministry of Finance is still not strongly committed to this.

“*The Ministry of Health and Ministry of Interior are aware of the constraints, but the Ministry of Finance still has to follow the recommendations and has to improve the financing of RAMED*” (Informant 16).

“*Now, in Morocco, to make a decision you are obliged to justify it by studies of recommendations. For example, when we recommended revising the eligibility criteria, the Ministry of Interior followed*” (Informant 4).

### Variation of learning development along the policy stages

We noticed that the learning process that was developed along the policy stages was different from one stage to the next; these differences have been summarised in Table 2.

**Table 2:** Evolution of organisational learning according to each stage of the policy

	Leadership that reinforces learning	Environment supportive to learning	Purposeful learning processes	Levels of learning
Agenda-setting and policy formulation	The leadership valued learning, with a focus on high strata of the public administration, through the inter-ministerial committee chaired by the Prime Minister	Persistence of a hierarchical administrative culture, with openings to technical participatory processes such as group work	Structured meetings, study tours to other countries, testing ideas in the field; however, many of these processes were not systematised and generated mainly tacit knowledge	Restricted to the individual level, mainly the committee members
Policy implementation	Emergence of leadership at the regional level with a multisectoral action. National leadership continued to value learning	The dynamic of group work extended to regional and local levels, with a focus on operational issues. Openness to ‘outsiders’ (national and international meetings). Hierarchical logic still present, but maybe less than before the policy	Structured meetings at local, regional and national levels. Experimentation through a pilot project. Development of an information system for follow-up of the policy implementation. Training, study visits, yet absence of a systematic approach to knowledge management	Learning occurred at group and team levels, mostly thanks to the work around guidelines and procedures
Policy evaluation	A central role is entrusted to the National Observatory of Human Development (ONDH)	Sustained effort to organise meetings and discussions including all departments involved in the RAMED policy	Organisation of meetings and workshops. The evaluation report is shared on the website of the ONDH	As for the Ministry of Health, learning mainly at the individual level (especially those involved in the evaluation)

### Limitations to developing organisational learning in each stage of the RAMED policy

As we have seen, there were plenty of positive interactions between the RAMED policy and the health system learning capacities. Still, our informants highlighted several limitations that prevented the achievement of optimal levels of organisational learning. Table 3 summarises the main limitations that hampered the development of organisational learning for each of the stages of the RAMED policy.

**Table 3:** Limitations of learning by stage and by blocks of learning

	Leadership that reinforces learning	Environment supportive to learning	Purposeful learning processes
Agenda-setting and policy formulation	• Continued influence of the hierarchical structure of public administration • Sometimes a top-down approach in decisions	• Difficulty in expressing all the points of view • Learning more at the individual level with weak organisational learning • The sharing was not for all aspects (some retention among departments)	• Practical processes were not systematised • Most developed knowledge was tacit • The learning agenda was episodic and ephemeral, and not integrated into the routine of organisations • Problems storing the knowledge for further use
Policy implementation	• Weak autonomy of hospital directors • Lack of resources to encourage learning at the local level • Continued influence of the hierarchical structure of the public administration	• Weak integrated information system • Weak sharing with other departments at regional level • People were not reassured enough to express their opinion regarding the design of guidelines and regulations • Openness to expressing ideas depended on the profile of the manager • There were overlapping roles of entities involved in RAMED (conflicts)	• Learning processes were quite ephemeral • Unshared reports (lack of platforms for sharing) • The practical processes of learning were not systematic (lack of systematic knowledge management strategy)
Policy evaluation	• The Ministry of Finance did not adopt the recommendations of the evaluation to increase resources for health	• Participation in the evaluation was limited to a few persons from the Ministry of Health, not a large participation in the discussion of recommendations	• Weak translation of the evaluation’s recommendation to action in the field for the implementation of RAMED

## Discussion

The main findings of this study are, firstly, that learning did exist in the policy process, although this was not well structured. Secondly, the actors involved in the RAMED policy acknowledged the importance of organisational learning but highlighted the lack of knowledge management mechanisms that could have made the learning more efficient. Thirdly, we have documented the fact that written materials on the RAMED policy were rather sparse; the fact that we had to rely largely on interviews indicates that Morocco lacks mechanisms to transform the abundant tacit knowledge into explicit knowledge through documentation. Fourthly, the RAMED benefited from the core team that initiated the policy formulation staying involved in the implementation; this helped the policy to benefit from the tacit knowledge accumulated through the whole process. Finally, the RAMED case (especially the lag between some decisions and actions and the very long duration of the pilot experiment) reminds us that, although learning is important in health policies, it is not sufficient – other conditions like resources or political willingness determine the results of the policy.

Our results are consistent with findings from previous research in Morocco [7]. There may be some progress, but the process of health policy design in Morocco remains characterised by verticality in the decision-making imposed by the rules of hierarchy. Early work on learning in public organisations highlighted that decision-making authority could be positively related to improved organisational learning [51]. In their study in Morocco, Blaise et al. [52] highlighted the existence of two conflicting logics in the Moroccan health administration, namely a normative logic, in line with public policy implementation on the one hand, and a creative logic responsive to emerging needs on the other. At that time, the latter was best addressed from outside the command and control of the line ministry (for instance, through projects). Blaise et al. [52] mentioned the improvement and evolution of scientific guidance in the Moroccan health system. Our findings confirm this positive evolution and show that the hierarchy has valued learning not only because it was an objective as such, but also – and maybe more – because it has emerged as a means of positioning oneself and climbing the administrative ladder. Although at the beginning of the RAMED policy learning was not planned and did not concern everyone in the health organisations, the policy process had a positive effect later on, spreading a culture of learning in support of health policies.

We found the policy implementation phase to be the most important phase. It is the one where the learning was developed in a more structured and scalable manner to reach the decentralised level. Even if not all the possible opportunities from the pilot project were seized (for instance, some of the lessons from the pilot region could have been better used for other regions), experimentation was key to prepare the scale-up of the RAMED. This is consistent with findings from a study of a similar scheme targeting the poorest in Cambodia [20] or with experiences of other health financing strategies [53]. The decentralised way of managing the implementation of RAMED through the pilot experiment helped to favour learning at the decentralised level. This finding is consistent with a study in South Africa emphasising that a centralised, mechanistic structure tends to reinforce past behaviours, whereas an organic, more decentralised structure tends to allow shifts of beliefs and actions [54]. The same study reported that hierarchy could be a barrier to organisational learning, and a lack of good leadership was mentioned as an impediment to organisational learning. A study in Burkina Faso reminds us that leadership is indeed crucial – if there is limited interest among national level decision-makers in the worst off, multiple efforts with knowledge processes may be made in vain [19].

Our assessment is that the RAMED policy evaluation contributed to some extent to giving factual data a stronger place in the decision-making at different levels of the health system in Morocco. However, this is an aspect which still needs much more reinforcement, as evidenced in our cross-sectional study [7]. A strong point is that most of the evaluation was done by an independent national observatory, which helped to gather different actors in the learning dynamic.

One fact mentioned was the limited effectiveness of learning when many other problems are not solved. Indeed, the lack of autonomy of hospitals impacted the effectiveness of learning in contributing to performance improvement. The learning dynamic thus missed its objective while arriving at the last link in the process, which is developing learning at the operational level. We conclude that learning is important, but not sufficient – resources, autonomy and power delegation should follow, too.

This research focuses on an aspect of the policy process that has received limited attention so far; therefore, our study has certain inherent limitations. We should be cautious about ascertaining causalities. Herein, we based the most important part of our information on interviewees’ opinions. There is maybe some influence of the power of hierarchy on our interviewees to express their opinions freely. To reduce the effect of this, we reassured participants on the ethical aspects of the study and tried to create a trusting environment during the interviews. The structure of the questionnaire also helped to triangulate and validate the information obtained. Recall bias could also be a limitation, but this was reduced by choosing people with a long-term involvement in the policy so that they could easily remember details. Finally, the study benefited from the fact that the first author, through his past and present positions in the MoH, has observed some of the reported processes.

Through this study, we hope to have shown the relevance of dedicating more attention to the issue of learning during a UHC policy process. More research work is needed for learning in health systems [8]. We see two priorities; one is to accumulate case studies like this one. This will allow, among other things, (1) a better understanding of the emergence and effectiveness of leadership supportive of learning; (2) identification of the best strategies when organisational cultures are not necessarily supportive of change; (3) appreciation of the strengths and weaknesses of different knowledge management approaches and techniques, including the exploitation of scientific and operational knowledge; and (4) better understanding of how a learning dynamic created by a specific political process can lead to more structural transformations in public health administrations. The second priority would be to better anchor this empirical knowledge in analytical frameworks that help us to establish causalities. We hope this article and the framework we have developed will inspire other researchers.

## Conclusion

This study has shed light on the strengths and weaknesses of organisational learning and knowledge management in the Moroccan health system as a necessary condition to improve organisational learning. From study of the RAMED policy, it emerges that this policy’s actions contributed to developing learning in the MoH and its different levels. Further, the policy actions benefited from the production of this learning through the design of the policy and the implementation tools. However, there was a discontinuity in terms of the pace of learning and conditions of learning after the pilot experiment. Therefore, the learning at the operational level has not contributed to achieving the best performance levels to improve the service for the population.

It is thus not sufficient to develop learning mechanisms, but rather we need to put in place conditions that will create learning leverage to improve the service. It is also clear that, by improving organisational learning without improving the other aspects that determine health organisations’ functioning (financing, human resources, autonomy, etc.), the effect of organisational learning will remain limited. For better organisational learning for UHC, countries have to invest in knowledge management entities to facilitate the sharing of knowledge on a large scale. Managers of health services need to have training on the role of learning in improving health system performance and increase their awareness of the importance of the practical process of learning. The use of digitalisation will better enhance the sharing of the vision, but also allows the creation of communication between operational and central levels of the MoH. In order to encourage dialogue and collaborative work, the MoH could launch a series of forums for discussion as communities of practice that have to be linked to the decision-making process.

We hope that this study will draw the attention of decision-makers to the importance of investing in a structured manner in learning for UHC. Use of the organisational learning framework in a learning organisation suggests directions for future actions to improve learning in health organisations – it is a matter of supportive leadership, a culture favourable to learning and appropriate learning processes. Some of these are in place in Morocco, but there is still some work to be done in this direction.

## Additional file

Additional file 1: Timeline of RAMED policy. (DOCX 43 kb)

## Abbreviations

AMO: Assurance Maladie Obligatoire
LMICs: low- and middle-income countries
MoH: Ministry of Health
RAMED: Régime d’Assistance Médicale
UHC: universal health coverage
