# Supplementary material for: The place of learning in a universal health coverage health policy process: the case of the RAMED policy in Morocco
Source: Health Res Policy Syst. 2019 Feb 21;17:21. doi: 10.1186/s12961-019-0421-6 (PMC6383252; doi:10.1186/s12961-019-0421-6)
Supplement: Supplementary file 1 — Timeline of RAMED policy. (DOCX 43 kb) [file 12961_2019_421_MOESM1_ESM.docx]

**Timeline of RAMED policy**

1960

1976

1998

2002

2005

2008

2011

2012

**Participation of communes for health services of their poor**

**First draft of the medical coverage law**

**Adoption of the law 65-00 of the basic medical coverage in Morocco**

**Implementation of the pilot experiment of RAMED**

**Evaluation of the pilot experiment of RAMED**

**Generalization of RAMED**

**Political dynamic**

**Transition government, socialists of the opposition**

**Creation of a high national committee to prepare a draft on basic medical coverage lead by the prime minister 96**

**Social movement of February 20 (Arab spring)**

**Reform of the constitution, art 31 right to health for the first time**

**First government after Arab spring under the new constitution**

**Creation of the National Angency of the health insurance (ANAM)**

**Health reform dynamic**

**Restructuring the health system and the reform of primary health care**

**Hospitals reform Funding Project for Health Sector Management (PFGSS projects), World Bank 76 million US$**

**The Support Program for Regionalization, Deconcentration and Strengthening of Basic Health Care (REDRESS et REDRESS p), 60 million euros, EU**

2016

**Evaluation of RAEMD (ONDH)**

**Hospitals reform, Santé Maroc III 140 million d’euros), UE**

**Support Project for Health Sector Management (PAGSS). 26 million euros, EU**

**SUPPORT PROGRAM OF THE REFORM OF BASIC MEDICAL COVERAGE PHASE I, 50 million euros, UE**

**SUPPORT PROGRAM OF THE REFORM OF BASIC MEDICAL COVERAGE PHASE II, 43 million euros, UE**

**SUPPORT PROGRAM OF THE REFORM OF BASIC MEDICAL COVERAGE PHASE III, 52 million euros, UE**

**Creation of an interministerial committee lead by the chief of government**

**Med 6 became king of Morocco 99**

**Development of application regulations of de compulsory health insurance (AMO)**

**Development of application regulations of the scheme for the poor (RAMED)**

**Free medical assistance (indigence Certificate)**

**Agenda setting of RAMED**

**Formulation of RAMED**

**Implementation of RAMED**

**Evaluation of RAMED**

**Medical coverage dynamic**

**The parliament refused the first draft of law 95, it excluded the poor**

**King Hassan II announces the principles of the medical coverage 93**

**Social dialogue included basic medical coverage**
